# Inflammation of the Bladder and Urinary Fever

**Published:** 1898-12

**Authors:** 


**Inflammation of the Bladder and Urinary Fever.** By C. Mansell Moullin, M.D. Pp. vii., 156. London: H. K. Lewis. 1898.

No better example could be found of the influence which bacteriology has exercised on the development of surgery than this book now before us. In it we find that the essential condition in the pathology of inflammation of the bladder is septic infection. Subsidiary causes are described, but they are said to be powerless without the pathogenic organisms, and Mr. Mansell Moullin affirms that there is no suppuration in the bladder without their presence and that all forms of cystitis are suppurative. The idea that mucus is secreted in the bladder he regards as erroneous, for he states that there are no mucous glands in its lining membrane, and that the ropy material we so often find in the urine in cystitis is simply pus altered by an alkaline condition of the urine.

Four distinct forms of urinary fever are described, and they are all traced to septic organisms in the urine, and the other theories as to the origin of this condition are set aside; but we must say the author fails to fully convince us of the truth of his views, though his doubt that the complete withdrawal of a large quantity of residual urine is able of itself to start any form of so-called urinary fever is one which seems very reasonable.

The portion of the work which treats of the sterilisation of catheters is very valuable to the practical surgeon, but he might be discouraged by the many difficulties raised. The mode of entrance of pathogenic organisms into the bladder is fully considered, and the cause of cystitis in women after operation, necessitating the recumbent position (although no catheter is passed), is discussed.

The chapter on tuberculous disease of the bladder is perhaps rather out of place in this work; but we are glad it has been inserted, as it is well worth careful study.

We cannot say that this book is written in such a way that the reader is led on from fact to fact, until he cannot fail to see a definite line of reasoning throughout it; but it is a very suggestive and instructive book, and places before the reader views on the relation of micro-organisms to inflammation of the bladder and urinary fever which he may have missed in recent periodical literature or have found inaccessible in the writings of foreign surgeons and bacteriologists.

